# Effects of salmon cartilage proteoglycan on obesity in mice fed with a high‐fat diet

**DOI:** 10.1002/fsn3.2685

**Published:** 2021-12-20

**Authors:** Shouhei Hirose, Krisana Asano, Seiyu Harada, Tatsuji Takahashi, Eriko Kondou, Kenichi Ito, Arunasiri Iddamalgoda, Akio Nakane

**Affiliations:** ^1^ Department of Biopolymer and Health Science Hirosaki University Graduate School of Medicine Hirosaki Japan; ^2^ Department of Microbiology and Immunology Hirosaki University Graduate School of Medicine Hirosaki Japan; ^3^ Department of Healthcare Dydo DRINCO, Inc. Osaka Japan; ^4^ Department of Research and Development Ichimaru Pharcos Co., Ltd. Motosu City Japan; ^5^ Department of Nursing and School of Health Sciences Hirosaki University of Health and Welfare Hirosaki Japan; ^6^ Present address: Division of Microbiology National Institute of Health Sciences Kawasaki Japan

**Keywords:** high‐fat diet, leptin, lipid metabolic enzymes, obesity, proteoglycan

## Abstract

This study investigated the effects of salmon nasal cartilage proteoglycan (PG), which shows anti‐inflammatory properties, on obesity induced by high‐fat diet (HFD) in a mouse model. Mice were fed either a HFD or normal diet (ND), with or without PG, for 8–12 weeks. After 12 weeks, the body weight of mice fed with PG‐free HFD was 54.08 ± 4.67 g, whereas that of mice fed with HFD containing PG was 41.83 ± 4.97 g. The results suggest that the increase in body weight was attenuated in mice fed with HFD containing PG. This effect was not observed in mice fed with ND. The PG administration suppressed the elevation of serum lipids (the level of serum lipids ranged between 54% and 69% compared to 100% in mice fed with PG‐free HFD) and the upregulated mRNA expression of sterol regulatory element‐binding protein‐1c (SREBP‐1c), which is a transcription factor that acts as a master regulator of lipogenic gene expression in the liver (the expression level was 77.5% compared to 100% in mice fed with PG‐free HFD). High leptin levels in mice fed with PG‐free HFD were observed during fasting (average at 14,376 ng/ml), and they did not increase after refeeding (average of 14,263 ng/ml), whereas serum leptin levels in mice fed with HFD containing PG were low during fasting (average of 6481 ng/ml) and increased after refeeding (average 13,382 ng/ml). These results suggest that PG feeding has an anti‐obesity effect and that the regulation of SREBP‐1c and leptin secretion play a role in this effect.

## Graphical abstract

Ingestion of proteoglycan (PG) in dry‐feeding form attenuated the increase in body weight of high‐fat diet‐fed mice. Ingestion of PG in dry‐feeding form reduced the levels of total cholesterol, high‐density lipoprotein cholesterol, triglycerides, and low‐density lipoprotein cholesterol in serum and the mRNA expression of lipogenic regulator, sterol regulatory element‐binding protein‐1c. Ingestion of PG in dry‐feeding form reduced leptin level during fasting and increased the serum leptin after refeeding.

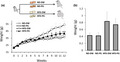

## 1 INTRODUCTION

Obesity is a lifestyle‐related disease, including type 2 diabetes, cardiovascular disease, hypertension, and fatty liver disease (Clemente et al., 2012; Kita et al., 2019). Furthermore, it is the fourth leading cause of mortality worldwide (Foreman et al., 2018; Kleinnert & Horton, 2019). It is important to note that adipose tissue is not only an energy‐storage organ, but also an endocrine and immunologic organ, secreting factors that modulate the function of different cells and tissues/organs (Dandona et al., 2004). Inflammatory cells, including T‐helper 1 (Th1) cells, Th17 cells, and macrophages, accumulate in the adipose tissue of obese hosts (Winer et al., 2009). Excess production of proinflammatory adipocytokines and decreased expression of anti‐inflammatory adipocytokines in obesity trigger systemic metabolic dysfunction and cardiovascular disease (Lackey & Olefsky, 2016; Nakamura et al., 2014).

Proteoglycan (PG) is a complex of glycohydrates consisting of core proteins with one or more covalently attached glycosaminoglycan chains. In cooperation with collagen, fibronectin, and laminin, PG has been shown to be involved in cellular proliferation and adhesion (Danen & Yamada, 2001). Structural information on PG extracted from salmon nasal cartilage has been reported (Kakizaki et al., 2011). Based on the deduced amino acid sequence, it was identified as an aggrecan lacking the keratin sulfate domain, which is commonly present in mammalian aggrecan. We have previously demonstrated that salmon cartilage PG can suppress inflammatory responses (Sashinami et al., 2006). In addition, daily oral administration of PG attenuates the severity of experimental inflammatory colitis (Mitsui et al., 2010), autoimmune encephalomyelitis (Sashinami et al., 2012), collagen‐induced arthritis (Yoshimura et al., 2014), skin allograft rejection (Asano et al., 2017), and type I allergy (Ono et al., 2018). Moreover, our previous study demonstrated that oral administration of PG in drinking water provided ad libitum improves hyperglycemia and insulin sensitivity in high‐fat diet (HFD)‐induced obesity in mice, but the effect of PG on body and adipose tissue weights was not observed (Hirose et al., 2017). It is possible that ingested PG potentially contributes to immunological homeostasis of the host by improving the composition of the gut microbiota (Asano et al., 2013).

In this study, we investigated the effects of ingestion of PG‐containing diets on HFD‐induced obesity in mice. We demonstrated that PG administration suppresses weight gain in HFD‐fed mice by modulating lipid metabolism and leptin secretion.

## 2 MATERIALS AND METHODS

### 2.1 Animals and diets

Female mice were used in our study for the application of PG in obese women. Six‐week‐old female Jcl/ICR mice were purchased from CLEA Japan, Inc., and maintained in a temperature‐controlled room (22°C) on a 12‐h light–dark cycle at the Institute for Animal Experimentation, Hirosaki University Graduate School of Medicine. Mice were either fed HFD (High Fat Diet 32) or received continuous normal diet (ND; CE‐2). Their nutritional composition is presented in Table 1. Both diets were purchased from CLEA Japan, Inc. All animal experiments were performed in accordance with the Guidelines for Animal Experimentation of Hirosaki University. All experiments on the mice were approved by the Committee on the Ethics of Animal Experimentation of Hirosaki University (permission number M10003).

**TABLE 1:** Nutritional composition of diets

Nutritional composition	Content (%)	
	ND	HFD
Moisture	9.1	6.2
Protein	24.8	25.5
Fat	4.6	32.0
Fiber	4.6	2.9
Ash	7.0	4.0
Nitrogen‐free extract	49.9	29.4
Energy (Kcal/100 g)	340.2	507.6

Abbreviations: HFD, high‐fat diet; ND, normal diet.

### 2.2 PG administration

**FIGURE 1:**
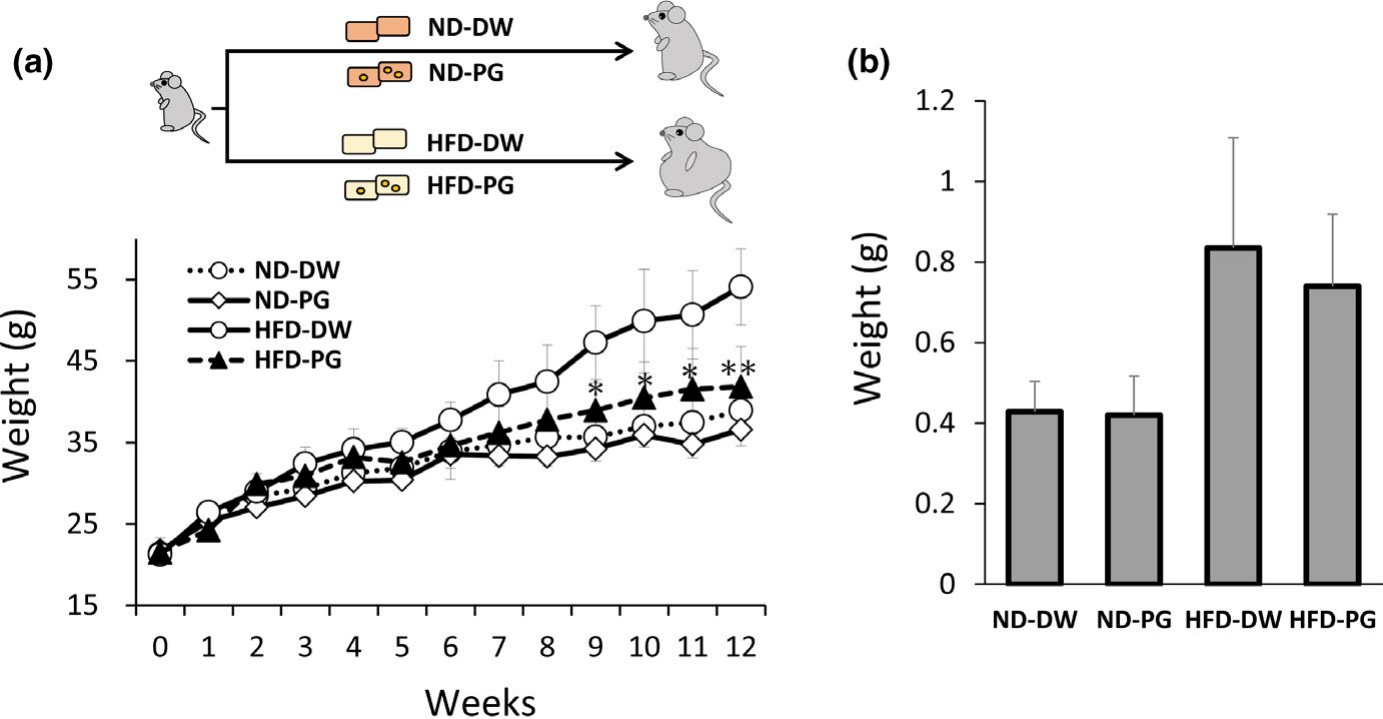
Effect of proteoglycan (PG) ingestion on body and adipose tissue weight in normal diet (ND)‐fed mice and high‐fat diet (HFD)‐fed mice. Six‐week‐old ICR mice were fed ND or HFD with or without PG for 12 weeks. Body weight was measured once a week (a). Adipose tissue was obtained from mice at 12 weeks of ingestion (b). Eight mice were used in each group. *p* value was less than *.05 and **.01, respectively

**FIGURE 2:**
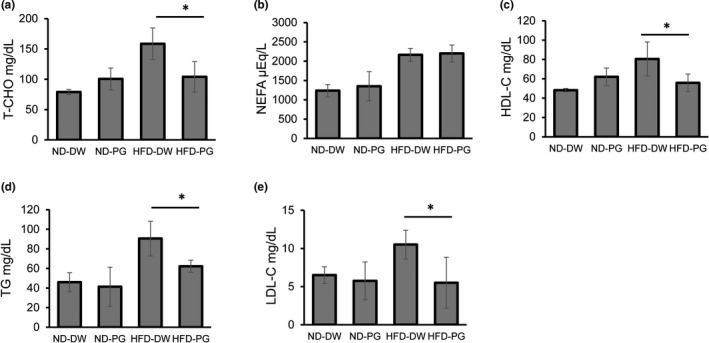
Effect of proteoglycan (PG) ingestion on serum lipid in normal diet (ND)‐fed mice and high‐fat diet (HFD)‐fed mice. Six‐week‐old ICR mice were fed ND or HFD with or without PG for 8 weeks. Serum specimens were obtained after fasting for 18 h. Concentrations of total cholesterol (a), non‐esterified fatty acid (NEFA) (b), high‐density lipoprotein cholesterol (c), triglycerides (d) and low‐density lipoprotein cholesterol (e) were determined. Eight mice were used in each group. *p* value was less than *.05

**FIGURE 3:**
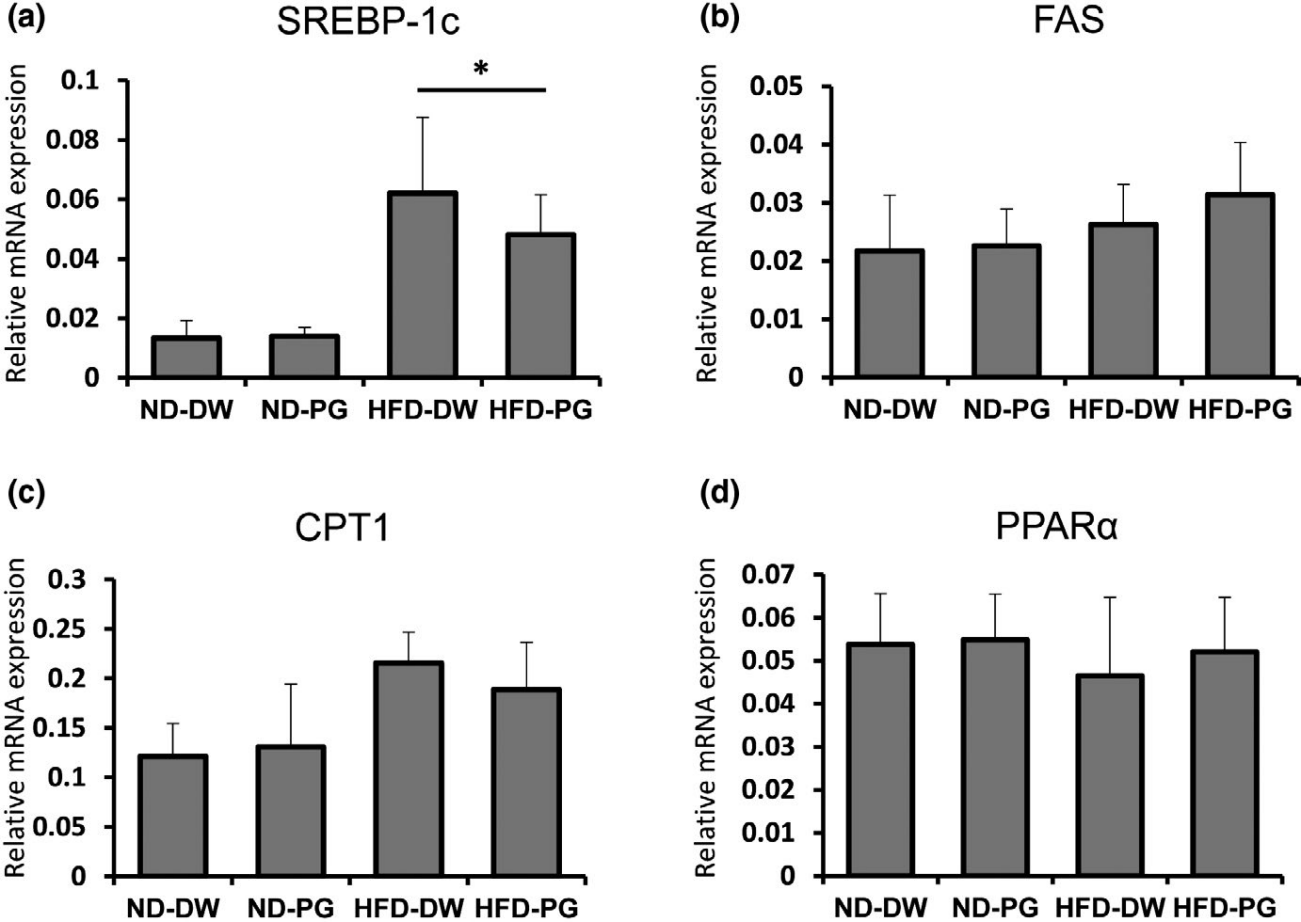
Effect of proteoglycan (PG) ingestion on lipid metabolic enzymes in liver of normal diet (ND)‐fed mice and high‐fat diet (HFD)‐fed mice. Six‐week‐old ICR mice were fed ND or HFD with or without PG for 8 weeks. Livers were obtained after fasting for 18 h. The mRNA expression of sterol regulatory element‐binding protein‐1c (a), fatty acid synthase (b), carnitine palmitoyltransferase 1 (c) and peroxisome proliferator‐activated receptor α (d) was estimated by real‐time quantitative reverse transcription‐polymerase chain reaction. Eight mice were used in each group. *p* value was less than *.05

**FIGURE 4:**
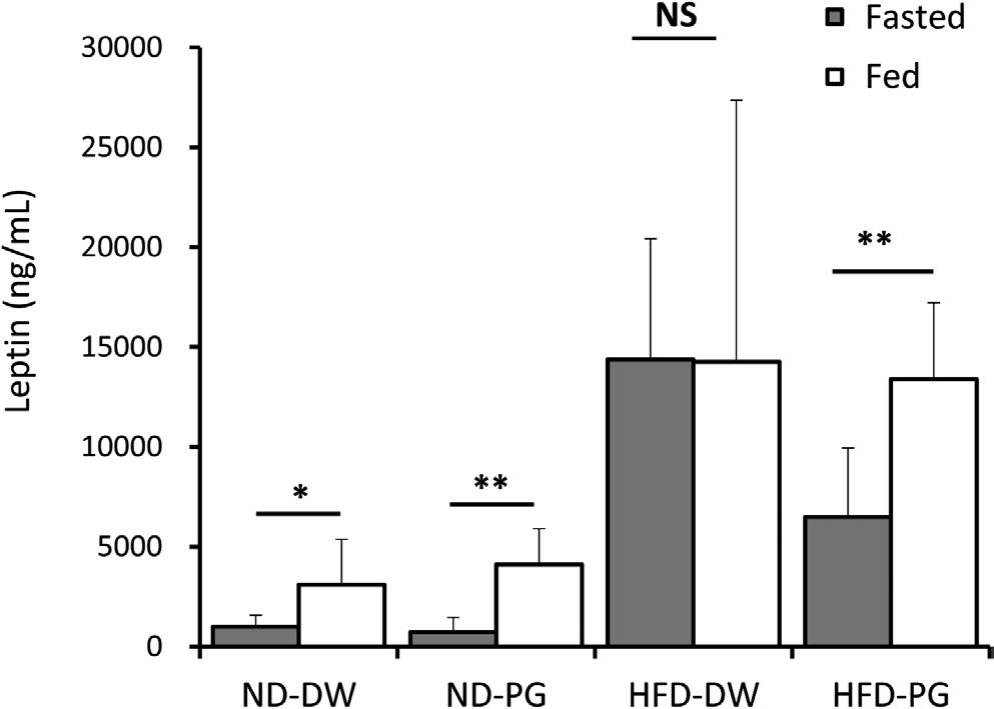
Effect of proteoglycan (PG) ingestion on leptin secretion in normal diet (ND)‐fed mice and high‐fat diet (HFD)‐fed mice. Six‐week‐old ICR mice were fed ND or HFD with or without PG for 8 weeks. Mice were fasted for 18 h and then given ND or HFD for 30 min. Serum specimens were collected before and after refeeding, respectively. Leptin concentrations in sera from before feeding (fasted) and after refeeding (fed) were determined. Eight mice were used in each group. *p* value was less than *.05 and **.01, respectively. NS means “not significant”

Proteoglycan was extracted from salmon (*Oncorhynchus kata*) nasal cartilage slices, as previously reported by Majima et al. (2001). Briefly, frozen salmon nasal slices were dissolved in a solution of 4% acetic acid to extract PG. We used high‐performance liquid chromatography analyses (TSKgel G5000PWXL column; Tosoh Corporation) and differential refractive index detection by RID‐10A for the quantitative and qualitative analysis of PG. The PG purity was >99% (Tomonaga et al., 2017). The extraction mass yield of PG from the raw material was approximately 0.7%. Purified PG was provided by Ichimaru Farcos Co., Ltd. PG was dissolved in distilled water (DW) at a concentration of 5 mg/ml. The PG solution was then mixed with powdered HFD or ND at a final concentration of 0.25 mg/g. In control mice, DW was mixed with powdered HFD or ND. Food and water were provided ad libitum. Mice from the HFD‐ and ND‐fed groups consumed approximately 4 g of diet daily. Therefore, daily ingestion of PG was estimated to be 1 mg per mouse. The PG continued to be administered for 12 weeks. Body weights were measured weekly. Ovarian visceral adipose tissue was isolated and weighed at 12 weeks. Eight mice were used in each group for all experiments. Experiments were performed four times as in Figures 1 and 2, and three times as in Figures 3 and 4, to confirm reproducibility.

### 2.3 Serum lipid analysis

Mice were fed for 8 weeks and fasted for 18 h prior to blood collection. Blood supernatants were obtained as serum after centrifugation at 1500 × *g* for 10 min. Concentrations of total cholesterol (T‐CHO), non‐esterified fatty acid (NEFA), high‐density lipoprotein cholesterol (HDL‐C), triglycerides (TG), and low‐density lipoprotein cholesterol (LDL‐C) in serum were measured at SRL Inc.

### 2.4 Real‐time quantitative reverse transcription‐PCR (qRT‐PCR)

Mice were fed for 8 weeks and fasted for 18 h before liver harvesting. Total RNA from the livers was extracted using the TRIzol reagent (Invitrogen). Moloney murine leukemia virus reverse transcriptase (Invitrogen) and random primers (Takara Bio Inc.) were used to synthesize first‐strand cDNA from 1 µg total RNA. The primers used to amplify target genes, including sterol regulatory element‐binding protein‐1c (SREP‐1c), fatty acid synthase (FAS), carnitine palmitoyltransferase 1 (CPT1), and peroxisome proliferator‐activated receptor α (PPARα), are listed in Table 2. The following conditions were used to amplify the target genes: Taq DNA polymerase activation at 95°C for 5 min, 40 cycles of 30 s at 95°C, 30 s at 60°C, and 30 s at 72°C. No primer‐dimer conformation or nonspecific amplification was confirmed by dissociation curves. The detection threshold was kept constant (0.05) for all the data analyses. Gene expression was calculated from the threshold cycle (C_T_) normalized to the expression of glyceraldehyde 3‐phosphate dehydrogenase (GAPDH), as previously described (Hirose et al., 2017).

**TABLE 2:** Primers used to amplify target genes from cDNA

Gene	Primer	Sequence
SREP‐1c	Forward	5′‐GGAGCCATGGATTGCACATT‐3′
	Reverse	5′‐CCTGTCTCACCCCCAGCATA‐3′
FAS	Forward	5′‐GCTGCGGAAACTTCAGGAAAT‐3′
	Reverse	5′‐AGAGACGTGTCACTCCTGGACTT‐3′
CPT1	Forward	5′‐GCACTGCAGCTCGCACATTACAA‐3′
	Reverse	5′‐CTCAGACAGTACCTCCTTCAGGAAA‐3′
PPARα	Forward	5′‐CCTCAGGGTACCACTACGGAGT‐3′
	Reverse	5′‐GCCGAATAGTTCGCCGAA‐3′
GAPDH	Forward	5′‐TGAAGGTCGGTGTGAACGGATTTGG‐3′
	Reverse	5′‐ACGACATACTCAGCACCGGCCTCAC‐3′

### 2.5 Leptin assay

Leptin in sera obtained from mice that fasted followed by being fed was assayed using enzyme‐linked immunosorbent assay (ELISA) kits (Universal Biologicals Ltd.). Briefly, mice that were fed for 8 weeks were fasted for 18 h and then fed ND or HFD ad libitum for 30 min before collecting blood. Serum specimens were obtained as described in Section 2.3.

### 2.6 Statistical analysis

Data are expressed as mean ± standard deviation, and *p* < .05 from Dunnett's test analysis was used to determine the significance of the differences in body weight, serum lipids, and expression of lipid metabolic enzymes. A *t*‐test was used to analyze serum leptin levels.

## 3 RESULTS

### 3.1 PG attenuated the increase in body weight of HFD‐fed mice

The effect of PG ingestion on obesity was investigated. Six‐week‐old ICR mice were fed ND or HFD with or without 1 mg PG per day. This dose was selected because we have previously reported that 0.08–2 mg salmon PG had anti‐inflammatory effects in a dose‐dependent manner (Mitsui et al., 2010; Sashinami et al., 2006,2012). In addition, the severity of obesity‐induced inflammation and papain‐induced allergy in mouse models was significantly attenuated by daily ingestion of 1 mg PG (Hirose et al., 2017; Ono et al., 2018). The body weight of the mice was measured once a week for 12 weeks. The results showed that the increase in body weight was significantly attenuated in mice fed HFD containing PG, compared with the ones fed HFD without PG (Figure 1a). After feeding for 12 weeks, the weight of mice fed with PG‐free HFD was 54.08 ± 4.67 g, whereas that of mice fed with HFD containing PG was 41.83 ± 4.97 g. In contrast, the PG ingestion did not affect the body weight of ND‐fed mice. The body weight of mice fed with PG‐free and PG‐containing ND was 38.93 ± 1.01 g and 36.56 ± 1.98 g, respectively. Adipose tissue was collected and weighed 12 weeks after feeding. Although adipose weight was slightly decreased in mice fed with the PG‐containing HFD, the difference was not statistically significant (Figure 1b). These results suggest that PG suppresses 38.1% gain in weight in HFD‐fed mice within 12 weeks through a diet containing PG.

### 3.2 Effect of PG on serum lipids in HFD‐fed mice

To investigate the effect of PG ingestion on serum lipids, mice were fed ND or HFD with or without PG for 8 weeks, and serum specimens were obtained after fasting for 18 h. In comparison with mice fed with PG‐free HFD (100%), the levels of T‐CHO, HDL‐C, TG, and LDL‐C were significantly decreased in mice fed with PG‐containing HFD (65%, 69%, 68%, and 54%, respectively; Figure 2). In contrast, no significant difference in serum lipids was observed between mice fed with ND containing and not containing PG.

### 3.3 Effect of PG on the expression of lipid metabolic enzymes in the liver of HFD‐fed mice

To investigate the effect of PG ingestion on the expression of lipid metabolic enzymes in the liver, mice were fed ND or HFD with or without PG for 8 weeks. The livers were harvested after fasting for 18 h. The mRNA expression levels of SREBP‐1c, FAS, CPT1, and PPARα were estimated by real‐time qRT‐PCR (Figure 3). The expression of SREBP‐1c mRNA was significantly downregulated in mice fed with PG‐containing HFD (77.5% compared to 100% in mice fed with PG‐free HFD) but not in animals fed with PG‐free ND. The effect of PG administration was not observed in the expression of FAS, CPT1, or PPARα in either HFD‐ or ND‐fed mice.

### 3.4 Effect of PG on leptin secretion in HFD‐fed mice

Leptin is mainly secreted by adipocytes after food ingestion, and it inhibits the production of neuropeptides and appetite stimulators (Stephens et al., 1995; Zhang et al., 1994). Therefore, serum leptin concentrations were determined either after fasting for 18 h or 30 min after refeeding (Figure 4). Leptin concentrations were increased (by approximately 3–6 times) after refeeding of mice with and without PG‐containing ND. In mice fed with PG‐free HFD, a high leptin level (14,376 ng/ml) was observed during fasting, and this level was not changed by refeeding (14,263 ng/ml). In contrast, in mice fed with PG‐containing HFD, leptin levels during fasting were lower than those in mice fed with PG‐free HFD (6481 ng/ml), and leptin concentration was then elevated by more than two times after refeeding (13,382 ng/ml).

## 4 DISCUSSION

The present study demonstrated that PG suppressed weight gain in HFD‐fed mice but not in ND‐fed mice when PG was provided in a mixed diet. However, our previous study demonstrated that the effect of oral administration of PG in drinking water provided ad libitum was not observed in HFD‐ or ND‐fed mice (Hirose et al., 2017). It is assumed that this discrepancy may depend on different absorption rates between the liquid‐ and dry‐feeding forms. A previous study showed that the original size of salmon nasal cartilage PG, but not chondroitin sulfate (CS), was recovered from the colon lumen when rats were administered PG or CS twice a day, suggesting that PG is more resistant to degradation than CS in vivo (Ota et al., 2008). PG may be rarely absorbed by the gastrointestinal tract. Therefore, further investigation is required to address this discrepancy.

Serum lipid levels are obesity indicators. All five lipids in mice fed with PG‐free HFD were higher than those in ND‐fed mice (Figure 2). The levels of T‐CHO, HDL‐C, TG, and LDL‐C were significantly decreased by PG intake in HFD‐fed mice. Next, we investigated the effect of PG feeding on the expression of enzymes involved in lipid metabolism in the liver. A significant effect was not observed in the expression of PPARα, a transcription factor related to β fatty acid (Dreyer et al., 1993), or FAS, which is involved in the synthesis of fatty acids and TG (Wakil, 1989) by either HFD feeding or PG ingestion (Figure 3b,d). The expression of CPT1, a target gene for PPARα involved in β oxidation of fatty acids in mitochondria (McGarry & Brown, 1997), was upregulated by HFD feeding, but it was not affected by PG ingestion (Figure 3c). In contrast, PG supplementation significantly suppressed the SPEBP‐1c expression, which was upregulated by HFD feeding (Figure 3a). SREBPs are transcription factors belonging to the basic helix–loop–helix/leucine zipper family, which are known to be master regulators of lipid metabolism and adipocyte differentiation. There are three isoforms of SREBPs: SREBP‐1a, SREBP‐1c, and SREBP‐2 (Osborne, 2000; Osborne & Espenshade, 2009). Among them, caloric restriction induces a more marked upregulation of SREBP‐1c than either of the other two isoforms (Chujo et al., 2013). Hence, SREBP1c, a transcription factor, acts as a master regulator of lipogenic gene expression in the liver (Awazawa et al., 2009). SREBP1c increases the expression of lipogenic enzymes, such as acetyl CoA carboxylase‐1 (Chen et al., 2004). Accordingly, genetic ablation of SREBP1c prevents hepatic lipid accumulation in HFD‐fed mice (Yahagi et al., 2002). Based on these observations, downregulation of the SREBP‐1c expression is involved in the PG anti‐obesity effect. In contrast, PG did not affect the expression of all tested lipid metabolic enzymes in ND‐fed mice (Figure 3). This result is consistent with the observation that PG did not affect body weight or adipose tissue weight in these control mice (Figure 1).

Leptin is a product of the obese gene that is mainly secreted by adipocytes, and its levels in the white adipose tissue and plasma are related to the energy storage so that leptin increases in obesity and decreases during fasting (Zhang et al., 1994). Leptin binds to leptin receptors within the ventromedial hypothalamus, where it inhibits the production of neuropeptides, which stimulate food intake, and thus decrease food intake, increase energy expenditure, and reduce body weight (Friedman & Halaas, 1998; Halaas et al., 1995; Satoh et al., 1997). Leptin secretion was observed in fasted ND‐ and PG‐fed mice and ND‐ and PG‐free mice after refeeding (Figure 4). Parallel with obesity, high levels of serum leptin were maintained in mice fed with HFD‐ and PG‐free diets, in both the fasting and feeding stages (Figure 4). In contrast, the HFD‐ and PG‐fed mice showed lower leptin levels compared with HFD control mice during fasting, and serum leptin was increased after refeeding in HFD‐ and PG‐fed mice. Therefore, it is assumed that PG may improve leptin resistance‐induced obesity (Yadav et al., 2013). Adiponectin is another important factor secreted in adipose tissue. Adiponectin tends to increase with fasting, and its expression decreases with an increase in adiposity (Halberg et al., 2008). The present results indicate that regulation of leptin secretion is involved in the anti‐obesity effect of PG.

In conclusion, PG ingestion shows a potent anti‐obesity effect in mice with HFD‐induced obesity. So far, we have shown that the regulation of helper and regulatory T‐cells and cytokine production is the main mechanism of the PG effect. In this study, we also found that PG regulates lipid metabolism and leptin secretion. Our unpublished results show that PG can exert a synergistic anti‐obesity effect in combination with other anti‐obesity gradients. Therefore, PG is a potent candidate that may improve obesity through oral supplementation.

## CONFLICT OF INTEREST

The authors have declared that no competing interest exists.

## ETHICAL APPROVAL

All animal experiments were carried out in accordance with the Guidelines for Animal Experimentation of Hirosaki University. All mouse experiments were approved by the Committee on the Ethics of Animal Experimentation of Hirosaki University (permission number M10003).

## Data Availability

The data that support the findings of this study are available from the corresponding author upon reasonable request.
